# Non-ossifying fibroma: natural history with an emphasis on a stage-related growth, fracture risk and the need for follow-up

**DOI:** 10.1186/s12891-016-1004-0

**Published:** 2016-04-05

**Authors:** Georg W. Herget, David Mauer, Tobias Krauß, Abdelrehim El Tayeh, Markus Uhl, Norbert P. Südkamp, Oliver Hauschild

**Affiliations:** Department of Orthopaedics and Traumatology, Comprehensive Cancer Centre Freiburg (CCCF), University Medical Centre Freiburg, Hugstetterstr. 55, 79106 Freiburg i. Br., Germany; Comprehensive Cancer Centre Freiburg CCCF, University Medical Centre Freiburg, Hugstetterstr. 55, 79106 Freiburg i. Br., Germany; Department of Radiology, University Medical Centre Freiburg, Hugstetterstr. 55, 79106 Freiburg i. Br., Germany; Department of Diagnostic and Therapeutic Radiology, St. Josefs Hospital, Sautierstrasse 1, 79104 Freiburg, Germany

**Keywords:** Non-ossifying fibroma, Imaging, Stages - symptoms, Follow-up

## Abstract

**Background:**

The aim of this study was to assess and present the radiological morphology of the non-ossifying fibroma (NOF), to describe the life span according to the Ritschl-stages in an effort to determine critical stages with regard to pathological fractures and discuss the need for a follow-up.

**Methods:**

Reports of a consecutive series of 87 patients with 103 NOFs and a mean follow-up of 27 months were analysed according to the Ritschl-stages with regard to age at time of diagnosis, localisation, duration of stage and symptoms.

**Results:**

Mean patient age in our series was 20 years and lesions most frequently affected the long bones of the lower extremity. Nineteen lesions were categorized in stage A, 53 in stage B, 17 in stage C and 14 in stage D. Most lesions were detected incidentally. In six of ten clinically symptomatic patients with an average age of ten years a pathological fracture occurred, and four of them were located in the tibia. All of these were in stage B with a mean length of 44 mm, an average expansion in relation to the bone-diameter of 75 % in transversal and 87 % in sagittal plane. Duration of the stages was variable. In the critical stage B the mean was 21 months.

**Conclusion:**

The non-ossifying fibroma follows a characteristic radiomorphological course with variable duration of each stage. Stage B lesions were found to be at an increased risk of fracture, and the age range over which fractures occur was wide. No fractures were detected in the other three stages. Follow-up, including clinical survey and imaging, at six to twelve month intervals may therefore be considered in the case of larger stage B lesions until stage C is reached.

## Background

The non-ossifying fibroma (NOF) is a common entity of bone, which is histologically characterized by a benign fibroblastic proliferation admixed with osteoclast-type giant cells [1]. It is a non-neoplastic process and belongs to the group of developmental abnormalities [2].

The actual incidence of NOFs is unknown. It has been estimated that approximately 30 % of children have one or more undetectes lesions [3, 4]. Radiologically, it is a solitary, eccentric and lytic lesion in the metaphysis of a long bone and, often polycyclic in shape [3, 4].

Nearly all lesions can clearly be diagnosed using plain radiographs. MRI is rarely necessary and limited to selected cases (diagnostic difficulties, e.g.), while the use of CT scans is inappropriate in young patients considering the radiation exposure associated with this imaging modality.

The disorder is most often asymptomatic [2], the prognosis is good and spontaneous regression over the years is typical [5]. Nevertheless, there is a certain risk for pathological fractures that mainly seem to depend on size.

However, to the authors´ best knowledge, fracture risk has thus far not been associated with stages according to a classification system introduced by Ritschl et al. [5], which includes 4 stages. Stage A represents an eccentric lesion near the physis. Stage B lesions have a variable distance from the physis with thin sclerotic borders. Stage C lesions exhibit increasing sclerosis. And, stage D lesions show complete sclerosis.

The aims of the present study were therefore

- to assess and present the radiological morphology of the non-ossifying fibroma
- to quantify the life span of the different stages as described by Ritschl
- to identify risk factors for the incidence of a pathological fracture through the lesion, namely lesion size, localization, patient age and Ritschl stage
- to derive a recommendation for a follow-up regimen

## Methods

Radiographs of 87 patients (57 males and 30 females with a ratio of m:f = 1.9:1) with a total of 103 lesions were retrospectively analyzed. Eleven patients had two and one patient six NOFs. All lesions were treated at a single institution over a period of 15.8 years including follow-up visits.

The vast majority of diagnosis was based on characteristic radiographic findings in plain radiographs. MRI scans were used for diagnosis and assessment in patients that had received MRIs previous to the referral to the authors´ institution. Secondary MRIs were performed in those rare cases with ambiguous radiographs. All radiographs were evaluated by two experienced physicians specialized in bone lesions.

The radiographs were analyzed sequentially for location, shape and border definition with special attention to the presence of sclerotic changes. Finally, all lesions were categorized according to Ritschl [5, 6]: Stage **A**: Eccentric lesion in the cortex near the epiphyseal endplate, which is small, oval to slightly polycyclic in shape without a sclerotic border. Stage B: Lesions with variable distance from the epiphysis with polycyclic shape and thin but clearly sclerotic borders, thin cortex with occasionally protruding above the surface like the shape of an hourglass; no periosteal reaction. Stage C: Lesions with properties similar to stage B but with also exhibit increasing sclerosis, which typically start from the diaphyseal side. Stage D 1–3: Complete homogeneous sclerosis of the lesion (D1), disappearing lesion (D2) and disappearance of the lesion (D3).

After categorizing the NOF, the length as well as the expansion in sagittal and transversal diameters were measured on radiographs; MRI based measurements were performed using the sequence which showed the maximum of expansion. The results were usually given as average value and standard deviation.

## Results

Average patient age at time of diagnosis was 20 ± 12 years. The lesions were located in the distal femur (47x), the proximal tibia (32x), distal tibia (20x), proximal fibula (2x) and in the proximal femur (1x) and distal fibula (1x). The average length of lesions was 38 ± 21 mm; one lesion was excluded due to incomplete presentation on radiographs. Average expansion in relation to the bone diameter was 39 ± 21 % in transversal and 46 ± 23 % in sagittal plane. Note that twelve lesions were excluded from the sagittal plane calculation due to incomplete presentation on radiographs.

**Fig. 1:**
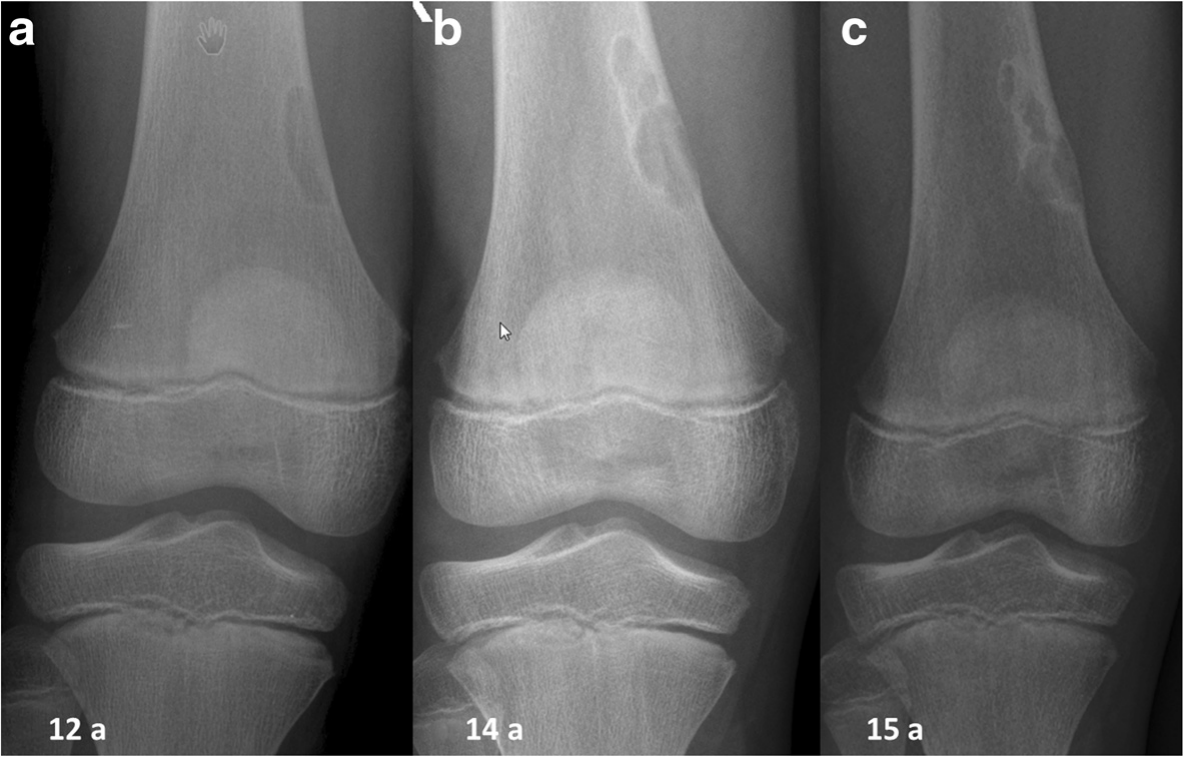
(**a**) A boy at the age of 12 years at first presentation. Anteroposterior radiographs showing a lytic lesion of the right distal femur representing a stage A lesion. (**b**) 2 years later the lesion was polycyclic in shape with clearly sclerotic borders (stage B). (**c**) At the age of 15 years there was evidence of ossification beginning at the diaphysis (stage c)

**Fig. 2:**
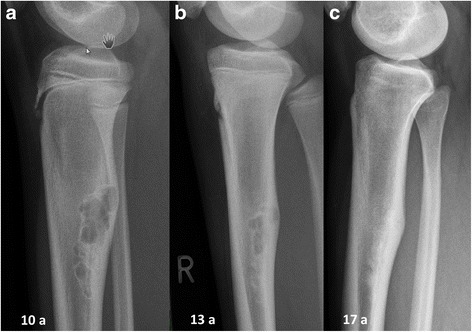
(**a**) A girl aged 10 years at the first presentation. Lateral radiographs of the right proximal tibia showing a typical NOF in stage B: distant from the epiphysis, polycyclic shaped and with clearly sclerotic borders. The cortex is protruding like the shape of an hourglass. (**b**) Three years later typical ossification beginning at the diaphysis is evident, representing a stage C lesion. (**c**) Complete homogeneous sclerosis of the lesion at the patient age of 17 years

The clinical data of stage A – D lesions (age at first presentation, length, transversal and sagittal expansion in relation to the bone diameter) are summarized in Table 1. Typical radiographic features of NOFs in different stages are shown in Figs. 1 and 2.

**Table 1:** Clinical Data of non-ossifying fibromas including age at detection, size in length, expansion in the transversal and sagittal diameter

	Stage A^a^	Stage B^a^	Stage C^a^	Stage D^a^
Number of patients	19	53	17	14
Average age (years)	13 ± 3.7	15 ± 5.7	20.4 ± 7.7	40.1 ± 12.4
Average length (mm)	21 ± 11	38 ± 16	47 ± 29	34.6 ± 15
Average transversal diameter expansion (%)	20 ± 9	46 ± 22	39 ± 12	30 ± 10
Average sagittal diameter expansion (%)	39 ± 14	56 ± 25	39 ± 12	32 ± 10

^a^Stage according to Ritschl [5]. Data provide average value and standard deviation

Ten of all patients with 11 lesions had clinical symptoms: pain on exertion, swelling and pathological fracture. Eight were classified as stage B (15 % of all stage B lesions) and 3 stage C lesions (18 %). Six of these 10 patients with a mean age of 10 years (range, 5–13 years) had a pathological fracture and all those lesions were in stage B. These lesions were located in the distal tibia (4x), proximal femur (1x) and proximal fibula (1x). Their average length was 44 ± 9 mm. The average expansion of the lesion was 75 ± 19 % and 87 ± 13 % of bone diameter in transversal and sagittal planes, respectively. The data are summarized in Table 2.

**Table 2:** Clinical data of patients with pathological fractures through non-ossifying fibromas

Case-Number	Age (Sex)	Location	Length (mm)	Expansion in transversal plane (%)	Expansion in sagittal plane (%)	Trauma
4	11 (m)	distal tibia	46	100	100	minimal trauma
13	11 (m)	distal tibia	43	58	68	minimal trauma fall after stumble
37	5 (w)	distal tibia	24	49	82	fall from a high with turning of the ankle joint
50	10 (m)	distal tibia	50	70	97	collision with opponent (football)
57	11 (m)	proximal fibula	47	100	100	fall from bicycle
103	13 (m)	proximal femur	52	74	73	spontaneous pain, no trauma
mean ± SD	10 ± 3	–	44 ± 9	75 ± 19	87 ± 13	–

*SD* standard deviation

Sixty-five of 103 lesions were followed over a mean period of 27 months (range, 1–167 months). During that time, 38 patients had one radiological examination or follow-up radiograph within one month of diagnosis. The stage change A → B → C was observed in18 patients (20 lesions) while two patients exhibited the stage change B → C → D. No stage-change was otherwise detected in the remaining patients of whom 8 had lesions that were in 8 stage A, 18 in stage B and 16 in stage C. Furthermore, 5 stage D lesions did not disappear – transition to stage D3 - during a mean observation time of 3 ± 2 months (range, 1–6 months). The duration of lesions in a single stage as well as stage-changes is summarized in Table 3.

**Table 3:** Duration of any single stage^a^ (A, B, C and D) in non-ossifying fibroma (NOF) with/without stage-change

	Stage A^a^	Stage B^a^	Stage C^a^	Stage D^a^
Lesions without SC (number)	8	18	16	5
Duration (months) mean ± SD	29 ± 16	21 ± 19	14 ± 9	3 ± 2
Lesions with SC (number)	4	15	2	1
Duration (months) mean ± SD	10 ± 4	21 ± 12	29 ± 9	49
Total number of lesions	12	33	18	6
Duration (months) mean ± SD	23 ± 16	21 ± 16	16 ± 11	11 ± 17

^a^Stage according to Ritschl [5]. *SC* stage-change, *SD* standard deviation

Growth of lesions was detected in stages A and B. Stage A growth had a mean 5 x 3 x 1.1 mm (SD ± 5/± 2.5/± 2.9 mm); three lesions were excluded from the sagittal calculation due to incomplete presentation. Stage B mean growth was on average 7 x 1.1 x 1.2 mm (SD ± 12.3/± 2.3/± 3.2 mm); one lesion was excluded from the transversal calculation and six from the sagittal calculation due to incomplete presentation on radiographs.

## Discussion

Non-ossifying fibromas belong to the most common lesions in bones, and multifocal occurrence is not uncommon. These lesions are no neoplasms and they are assigned to the group of developmental abnormalities. Aetiologically, an injury in the area of muscle attachment resulting in a focal, subperiosteal haemorrhage was discussed [3, 4].

Multifocal NOFs have been reported in patients with the Jaffe–Campanacci syndrome (multiple NOFs, café-au-lait spots, mental retardation, hypogonadism or cryptorchidism, ocular anomalies or cardiovascular malformations) [7–9] and in patients with Type 1 neurofibromatosis [2].

It is estimated that NOFs are present in about 30 % of children [1]. In other studies, Freyschmidt described an incidence of 1–2/100 in the first two decades [10] and Jundt a prevalence in 2.065 patients ≤ 20 years of 1.8 % [11]. A male predominance (m:f = 1.9:1) was found in the present series, which is in agreement with previous investigation [12].

While the majority of NOFs have been reported to develop in patients under the age of 15 years [13, 14], the mean age at diagnosis in our series was 20 years for all patients, 13 years in the stage A and 15 years in the stage B group, respectively, while the latter group represents the largest (53 patients).

Usually, NOFs are discovered incidentally on plain radiographs made for other reasons. In our investigation 89 % of patients were asymptomatic. In a few cases a NOF can cause pain and swelling, especially associated with athletic activity [15].

The vast majority of the lesions develop in the metaphysis of the long bones of the lower extremities [5, 15, 16]. As in other investigations [12, 17] we observed most lesions (79 %) in the region of the knee.

Characteristic radiographic appearance lends considerable certainty to the diagnosis [5, 18]. Typically, the lesion is lucent, the margins range from being densely sclerotic, or scalloped, to being hazy and indistinct, and the cortex may be thinned and in some cases it is expanded [19, 20]. The greatest length of the lesion tends to be in the long axis of the bone [19, 20]. Notably, when a NOF is detected by chance it causes, with surprising frequency, unjustified concern that leads to consultations in orthopaedic oncology clinics [21]. This may be attributed to the variability of radiological presentations: they are biologically active, may grow, and, in their involution phase, somewhat contrary to its name, gradually become filled with bone tissue [3, 5]. This results in different stages, which were subdivided in 4 groups (A-D) according to Ritschl [5, 6].

In our series, 19 patients with a mean age of 13 had a stage A lesion with a mean length of 21 mm. Fifty-three patients with a mean age of 15 had a stage B lesion with a mean length of 38 mm. Eight of these patients had clinical symptoms and the mean length of their lesions was 43 mm. Seventeen patients with a mean age of 20 had stage C lesions with a mean length of 47 mm. Three of these patients had clinical symptoms, the mean length of their lesions was 58 mm. And, 14 patients with a mean age of 40 had a stage D lesion with a mean size of 35 mm.

In terms of the age, similar age groups were described by Blaz with a mean age of 11 years in stage A, 16 years in stage B, 18 years in stage C and 23 years in stage D [3], with the latter differing from our results. This is explained by inclusion of stage D subgroups (D1-3; mentioned above) in our results.

Exclusively the stage B group (8 of 53 patients) had clinical symptoms. The lesion size was on average 43 mm in length and average expansion in relation to the bone diameter was 66 % in the transversal and 76 % in the sagittal plane. Six of the eight had a pathological fracture, while in these cases the average size of lesion was 44 mm in length, average expansion was 75 % and 87 % of the bone diameter in the transversal and sagittal planes, respectively. It should be mentioned that the patient with the 24 mm long lesion jumped down from a height of more than half of a meter with a consecutive turning of the ankle joint, which cannot be considered a “minimal trauma” as seen in the other patients. It is also remarkable, that four of six fractures were located in the distal aspect of the tibia.

In a review by Arata et al., 23 cases of pathological fractures through NOFs were described [22]. The average patient age was 12 years and all fractures except one were located in the lower extremity with ten in the distal tibia [22]. The percentage of bone occupied by NOF exceed 50 % in both planes (anterior-posterior and lateral), and the vertical length exceed thirty-three millimeters in all non-fibular lesions. They concluded, that lesions of that size should be monitored closely [22]. In contrast, in the series of Easley and Kneisl 59 % of cases of large NOF exceeded these measurements and did not fracture; the authors therefore suggested that the majority of patients with large NOFs can be monitored without intervention, as there is evidence to support spontaneous resolution of the majority of these lesions [23]. However, in both studies no correlation was performed with respect to the stage according to Ritschl, which may account for the different results.

In conclusion, patients with a stage B lesion have an increased risk of suffering a fracture. However, no fractures were found in stage A, C and D. In our series, fractured lesion lengths were on average 44 ± 9 mm long and expansion in relation to the bone diameter was on average 75 ± 19 % in transversal and 87 ± 13 % in sagittal plane. Furthermore, the location in the distal tibia seems to increase the risk of suffering a pathological fracture.

The fractures in our patients occurred at a mean age of 10 years (range, 5–13 years). Arata et al. described a mean age of 12 years (range 4–28 years) for patients who suffered a fracture in his own series and a mean of 12.8 years in several summarized series [22]. In another publication the average age of the fracture-group patients was 14.3 years (range 6.9–20.3) [23]. In summary, there is on the one hand a mean age with an increased incidence of fractures and on the other a wide age range over which fractures occur.

Although not specifically addressed in our study, it should be mentioned that fractures due to NOFs exhibit excellent healing potential [18, 22], which is frequently observed without bone grafting [18, 22]. But solid union can probably be obtained earlier with the addition of bone grafts [19]. And, unless curettage and bone grafting are performed, the lesion usually persists after healing of the pathologic fracture [18, 22].

Because fractures were shown to be stage–related while the initial lytic lesion became, over time, sclerotic and resolved, stage duration is of special interest. In our series stage A lasted 23, stage B 21, stage C 16 and stage D 11 months, on average. Lesions that underwent any kind of surgical intervention related to their NOF were excluded from these results. In a study by Rischl et al. the radiographic evolution was observed in 61 cases for more than a year and in 30 for less than a year with an average total observation for the 61 cases of 56.4 months (range, 15–192 months) [12]. He described a stage-change A → B for 7 lesions within a mean time of 19.7 months, A → C (1 lesion) within 80 months, A → D (4 patients) within a mean time of 69.2 months, B → C (9 lesions) within a mean time of 21.3 months, B → D (19 lesions) within a mean time of 50.7 months and C → D within am mean time of 45.4 months [12]. Sontag and Pyle noted an average lesion duration of 29 months [24], while Caffey reported the average duration to be almost 53 months [25]. In the series of Drennan et al. several lesions noted in childhood were without radiographic evidence at follow-up 10–19.5 years later [18].

It can therefore be summarized that, including our own results, the duration of every single stage is variable. Considering the fact that almost every patient included in our analysis lacks either the starting or the endpoint of the “critical” stage B we assume that the average duration of this stage is longer than 20 months. Considering solely stage B lesions (15x) that changed into stage C, we observed the average duration was 21 ± 12 months (range, 8–44 months).

With respect to these findings, and especially the risk of suffering a fracture, follow-up is recommended during stage B, which is very variable in duration. But note, that only six of 53 patients in stage B suffered a pathological fracture. For those that did, the size of the lesion appears to be important with larger lesions being more prone to resulting in fracture. The patients should therefore be advised to reduce or avoid strenuous activity in an effort to avoid fracture. And, these patients should be more closely monitored and follow-up is advised with an six to twelve month interval including clinical survey and imaging.

Limitation of the study: Appreciation for the limitations of this study is warranted. First and foremost this is a retrospective study. Owing to this and the benign nature of the lesion follow-up examinations were not available in about one third of patients. Nonetheless, two-thirds of the lesions could be followed over an average period of 26 months. Including the remaining patients provided additional epidemiologic data for reconsidering previously established stages, describing the age of detection, size of lesion and possible clinical symptoms.

As biopsy was generally not performed on these lesions the diagnosis was established based on radiological findings. We tried to compensate for this by having the radiographs reviewed independently by two experienced physicians.

Owing to the fact that follow-up assessments were available for few of the patients, the scope for statistical analyses on the development of NOF was limited. The analyses were therefore performed descriptively in order to generate hypotheses for further clinical investigations.

## Conclusions

Most non-ossifying fibromas were detected in childhood to late adolescence, were found incidentally and were clinically asymptomatic. They followed a characteristic natural course while the duration of any single stage (A - D) was variable. During stage B the lesions are at an increased risk of fracture; fractures occurred over a wide age range. They were mostly located in the distal tibia. The average length of fractured lesions was 44 ± 9 mm, the average expansion in relation to the bone diameter was 75 ± 19 % in transversal and 87 ± 13 % in sagittal plane, respectively. Hence, it is reasonable to pay attention to the duration of the stages, the localization and size of the lesion, rather than patient age at time of NOF detection. Follow-up might therefore be considered in the case of larger stage B lesions including clinical survey and radiographs at six to twelve months after diagnosis until stage C is reached.

### Ethics approval

Research was performed in accordance with the Declaration of Helsinki and has been approved by the local ethics committee (Ethics Committee Office, Albert-Ludwigs-University Freiburg, Engelberger Straße 21, 79106 Freiburg i. Br., Germany. Application number 202/14).

### Consent for publication

All patients gave written inform consent according to the university hospital standard.

### Availability of data and materials

The whole dataset supporting the conclusions of this article is are available by the corresponding author.

## Abbreviations

NOF: non-ossifying fibroma
WHO: World Health Organization
SD: standard deviation
